# Divergent Immune Responses to Minor Bovine Mastitis-Causing Pathogens

**DOI:** 10.3390/vetsci11060262

**Published:** 2024-06-07

**Authors:** Anyaphat Srithanasuwan, Noppason Pangprasit, Raktham Mektrirat, Witaya Suriyasathaporn, Phongsakorn Chuammitri

**Affiliations:** 1 Veterinary Science Unit, Faculty of Veterinary Medicine, Chiang Mai University, Chiang Mai 50100, Thailand; anyaphat.sw@gmail.com; 2 Department of Animal Sciences, Wageningen University, 6708 PB Wageningen, The Netherlands; 3 Akkhraratchakumari Veterinary College, Walailak University, Nakhon Si Thammarat 80160, Thailand; noppason.pa@wu.ac.th; 4 Veterinary Bioscience Unit, Faculty of Veterinary Medicine, Chiang Mai University, Chiang Mai 50100, Thailand; raktham.m@cmu.ac.th; 5 Research Center for Veterinary Biosciences and Veterinary Public Health, Faculty of Veterinary Medicine, Chiang Mai University, Chiang Mai 50100, Thailand; 6 Veterinary Academic Office, Faculty of Veterinary Medicine, Chiang Mai University, Chiang Mai 50100, Thailand; witaya.s@cmu.ac.th; 7 Research Center of Producing and Development of Products and Innovations for Animal Health and Production, Chiang Mai University, Chiang Mai 50100, Thailand; 8 Nagoya University Asian Satellite Campuses, Institute-Cambodian Campus, Royal University of Agriculture, Dangkor District, Phnom Penh 370, Cambodia

**Keywords:** non-aureus staphylococci and mammaliicocci, lactic acid bacteria, pathogen recognition, apoptosis, bovine neutrophil

## Abstract

**Simple Summary:**

Bovine mastitis can be caused by both bacterial and non-infectious agents. Well-established mastitis pathogens include *Staphylococcus aureus*, *Streptococcus agalactiae*, and *Streptococcus uberis*. Traditionally, non-*aureus* staphylococci and mammaliicocci (NASM), including *Staphylococcus chromogenes*, and lactic acid bacteria (LAB) were not considered major contributors to bovine mastitis. However, their role in subclinical mastitis is increasingly recognized. This study investigated the involvement of NASM and LAB strains (*Weissella paramesenteroides*) through the lens of bovine neutrophil responses. This study suggests that *S. chromogenes* and *Weissella* exhibit divergent immunomodulatory effects on bovine neutrophils via coordinated functional and gene expression alterations. These findings may necessitate a re-evaluation of their respective roles in bovine mastitis etiology.

**Abstract:**

Traditionally, non-*aureus* staphylococci and mammaliicocci (NASM) were not considered significant players in bovine mastitis. This study investigated the involvement of NASM (*Staphylococcus hominis* and *Staphylococcus chromogenes*) and lactic acid bacteria (LAB) strains (*Weissella paramesenteroides*) through bovine neutrophil responses. Bovine neutrophils displayed minimal apoptosis upon NASM and LAB challenge. Neutrophils expressed high TLR2 after challenge, but TLR6 expression varied and remained low in NASM pathogen recognition. Bovine neutrophils effectively engulfed and killed LAB, but their activity was significantly impaired against NASM. This was evident in *S. chromogenes*, where reduced TLR6 recognition and a weakened phagocytic response likely contributed to a lower bactericidal effect. Regardless of the bacteria encountered, intracellular ROS production remained high. *S. chromogenes*-challenged neutrophils displayed upregulation in genes for pathogen recognition (TLRs), ROS production, and both pro- and anti-apoptotic pathways. This response mirrored that of *Weissella*. except for *CASP9* and *BCL2*, suggesting these bacteria have divergent roles in triggering cell death. Our findings suggest that *S. chromogenes* manipulates bovine neutrophil defenses through coordinated changes in functional responses and gene expression, while LAB strains have a weaker influence on apoptosis.

## 1. Introduction

Bovine mastitis arises from both infectious (mostly bacteria) and non-infectious factors. Up to 95% of cases stem from bacterial invaders like *Staphylococcus aureus*, *Streptococcus agalactiae*, *Streptococcus uberis*, *E. coli*, and *Klebsiella pneumoniae*, triggering udder inflammation [1]. While major mastitis pathogens dominate headlines, non-*aureus* staphylococci (NASM) like *Staphylococcus chromogenes* are increasingly found in subclinical mastitis. Though dubbed “minor pathogens”, their true impact remains debated due to their presence in both healthy and infected quarters [2]. *S. chromogenes* leads the pack as the most common NASM isolates [3,4,5]. Among NASM, *S. chromogenes*, *S. simulans*, and *S. xylosus* all raise SCC, with *S. chromogenes* leading in causing persistent infections [6].

The rise of NASM in both human and veterinary medicine is concerning. This is due to the finding of antimicrobial resistance genes (*mec*A, *bla*Z), the high prevalence of virulent factors (DNAse, gelatinase, biofilm formation), and even the production of some staphylococcal enterotoxins [5,7,8,9]. Healthy udders are crucial for optimal milk yield and animal welfare [10]. While major and minor pathogens like NASM pose challenges, certain NASM like *S. chromogenes* offer benefits. This commensal from healthy cows inhibits mastitis-causing biofilm formation in *S. aureus* without harming bovine neutrophils [11].

Bovine lactic acid bacteria (LAB) show promise as a non-antibiotic approach to mastitis [12]. Many LAB strains strongly inhibit mastitis pathogens like *S. aureus*, *S. uberis*, and *S. agalactiae*, suggesting potential as probiotics for prevention and treatment [12,13]. Notably, some LAB strains colonized milk, adhered to teat canal cells, and even inhibited and co-aggregated with mastitis-causing bacteria [13]. LAB’s diverse weapons against mastitis include antimicrobial peptides (AMPs, e.g., enterocin A, bacteriocin), lactic acid, H_2_O_2_ (like in *Weissella cibaria* and *W. paramesenteroides*), surface adhesion, and protective biofilms from exopolysaccharides (EPS) [14]. These traits make them promising and safe alternatives for mastitis prevention and treatment [14]. While we have explored bovine neutrophil responses to major and minor mastitis bacteria [15], detailed interactions between minor pathogens, like *S. chromogenes* and LAB, and the host’s immune response remain scarce.

The dynamics of udder immunity and infection are multifaceted, influenced by factors such as the cow’s health status, environmental conditions, and the presence of subtle minor pathogens like NASM and LAB. Bovine udder immunity hinges on the early detection of invading pathogens. Toll-like receptors (TLRs) on neutrophils and macrophages act as sentinels, recognizing microbial components and triggering the immune response. Bovine neutrophils wield an arsenal of Toll-like receptors (TLRs), including TLR1, TLR2, TLR4, TLR6, TLR7, and TLR10, allowing them to recognize diverse pathogens. These receptors detect lipoproteins, peptidoglycans, and zymosan in Gram-positive bacteria and LPS in Gram-negative ones [16]. Unraveling how bovine neutrophils recognize “minor players” like NASM and LAB, especially *S. chromogenes*, holds the key to understanding udder immunity dynamics. Upon pathogen recognition, bovine neutrophils wage war with classical tactics (phagocytosis, ROS, RNI, AMPs, granule enzymes) [17], and subtle strategies (NETs, inflammatory mediators) for comprehensive udder defense unfold [15,18,19,20,21]. Adding another layer to their defense, neutrophils undergo programmed death (apoptosis) to limit NASM and LAB infections and resolve inflammation, revealing the intricate interplay between host and pathogen.

While the complex interplay between udder immunity, minor pathogens like NASM and LAB, and environmental factors is acknowledged, crucial details are missing. We lack a deep understanding of how bovine neutrophils—key players in udder defense—manipulate their responses to these bacteria to prevent infection. Elucidating their strategies is vital for developing effective interventions. This study aims to fill this gap by deciphering and investigating the pathogen recognition receptors that lead to specific response mechanisms and unraveling the intricacies of the immune resolution of neutrophils against minor pathogens.

## 2. Materials and Methods

### 2.1. Animals, Blood Collection, and Bovine Polymorphonuclear Neutrophil (PMN) Isolation

Blood samples were obtained from adult, non-pregnant Holstein Friesian cows (*Bos taurus*) maintained in good health at a smallholder dairy farm. These cows were housed in tie-stall barns with concrete floors. The cows were 3–7 years of age, weighed 350–400 kg, and had a body condition score of 3 to 3.5. They were in their second to fourth lactation periods. The diet provided consisted of a commercial meal concentrate (2.5 kg offered twice daily) and fresh roughage ad libitum. The roughage component included by-products such as corn silages, rice straws, and grass hay. Water was also available ad libitum.

For this study, a total of ten cows were used throughout two independent experiments (during the period June to September 2023), each using five cows. All cows were housed at local farms in Mae Wang district, Chiang Mai, Thailand. Using jugular venipuncture, about 40 mL of whole blood was obtained into a sterile syringe containing 10 mL of 1× acid citrate dextrose solution for bovine polymorphonuclear neutrophil (PMN) isolation within 2 h after blood collection. Bovine neutrophil isolation was performed as described [15]. The cell density was adjusted to approximately 3 × 10^6^ cells per mL. The conducted animal experiments were approved by the Animal Care and Use Committee (FVM-ACUC) under the reference number R16/2565.

### 2.2. Bacterial Growth Condition

Bovine non-*aureus* staphylococci and mammaliicocci (NASM); *Staphylococcus hominis* (SHO) and *Staphylococcus chromogenes* (SCH); lactic acid bacteria (LAB); and *Weissella paramesenteroides* (WPA), with antimicrobial activity against *S. aureus* or *Escherichia coli,* were selected as minor pathogens causing bovine mastitis for use in the entire experiment [15]. Bacteria (NASM) were grown on Tryptic soy agar plates (TSA, HIMEDIA, Mumbai, India) with 5% bovine blood for 24 h at 37 °C. *Weissella paramesenteroides* (WPA) was grown on Lactobacillus MRS agar (De Man–Rogosa–Sharpe), HIMEDIA) plates and incubated microaerobically at 37 °C for 24 h before use. The bacteria number was adjusted to approximately 10^8^ CFU/mL in the experiment before use.

### 2.3. Fluorescent Labeling and Opsonization of Bacteria

Live bacteria from Section 2.2 were grown to the log phase, suspended in a Hanks’ balanced salt solution (HBSS) (Sigma-Aldrich, St. Louis, MO, USA), and heat-killed at 70 °C for 60 min using a water bath (Memmert, GmbH, Schwabach, Germany). Heat-killed bacteria were resuspended at a 10^8^ CFU/mL density in 1 µg/mL Hoechst 33,342 (Invitrogen, Thermo Fisher Scientific) for SCH and WPA. Bacteria were fluorescently labeled for 30 min at 4 °C. Then, they were washed extensively with HBSS to remove the free dye, adjusted to 10^6^ CFU/mL with HBSS, and stored at 4 °C until use. Fluorescently labeled bacteria were opsonized with 10% heat-inactivated autologous bovine serum for 20 min at 37 °C before using in the phagocytosis assay.

### 2.4. Bovine Neutrophil Apoptosis Assay

Bovine neutrophils (1 × 10^5^ cells) were stimulated with live SHO, SCH, and WPA bacteria at the MOI of 10 in HBSS. The cells were then incubated for 45 min at 37 °C with 5% CO_2_. For viability analysis, cells were stained with a FITC Annexin V apoptosis detection kit (BioLegend, San Diego, CA, USA) and analyzed by a DxFLEX Flow Cytometer (Beckman Coulter, Brea, CA, USA) and FlowJo 10 (Treestar, Ashland, OR, USA) as described [15].

### 2.5. Analysis of Surface Microbial Associated Molecular Pattern (MAMP) of Bovine Neutrophils

Isolated bovine neutrophils (3 × 10^5^ cells) were stimulated with either live SCH or WPA bacteria at the MOI of 10 at 37 °C with 5% CO_2_ for 45 min. After incubation, cells were washed twice with ice-cold HBSS and pelleted. Cells were resuspended in 100 µL cell staining buffer (CSB) (composed of 1× PBS, FBS, and sodium azide) and an additional 100 µL of 2% Fc receptor blocker (composed of CSB and autologous serum) and incubated for 20 min at 4 °C. Next, anti-mouse TLR6 PE-conjugated monoclonal antibody (clone 418601, R&D systems) and Alexa Fluor^®^ 647 anti-mouse/human CD282 (TLR2) recombinant antibody (clone QA16A01, BioLegend, San Diego, CA, USA) were resuspended in CSB and added to cell pellets and incubated for 30 min at 4 °C. Cell pellets were washed with CSB and analyzed by flow cytometry. Mean fluorescence intensity (MFI) was then determined.

### 2.6. Measurement of Intracellular Reactive Oxygen Species (ROS)

Bovine neutrophils (3 × 10^5^ cells) were activated to produce ROS for 30 min with each live bacteria at the MOI of 10 in HBSS. Then, 10 µM H_2_DCF-DA (Invitrogen, Thermo Fisher Scientific) was loaded into each well to stain the intracellular H_2_O_2_. Cells were incubated in the dark for 15 min, then washed with cold HBSS. ROS-positive cells were analyzed by a flow cytometer [21].

### 2.7. Phagocytosis of Fluorescently Labeled Bacteria

The phagocytosis of fluorescently labeled bacteria (SCH or WPA) was assessed via flow cytometry. In brief, bovine neutrophils (3 × 10^5^ cells) were mixed with opsonized, Hoechst 33,342 labeled bacteria at the MOI of 10. Heat-killed bacteria with the omission of fluorescent labeling were used as controls. To promote the uptake of bacteria, the cell mixture was centrifuged at 1200 rpm for 3 min, and the bovine neutrophils were allowed to uptake the bacteria for 45 min at 37 °C, 5% CO_2_. After incubation, cells were quenched with 0.4% trypan blue and washed extensively with ice-cold HBSS before being analyzed by a flow cytometer.

### 2.8. Bacterial Killing and Spot Dilution Assay

The bactericidal activity of bovine neutrophils was assessed using a semi-quantitative MTT test to determine the percentage of bacterial viability. Isolated bovine neutrophils (3 × 10^5^ cells) were stimulated for 45 min with opsonized live bacteria (SHO, SCH, WPA1, and WPA2) at the MOI of 10. Internalized bacteria were released from neutrophils by sterile H_2_O lysis. A portion of the supernatant was saved for a subsequent spot dilution assay. Following lysis, TSB or MRS broth containing 2 μg/mL Thiazolyl Blue Tetrazolium Bromide (MTT, Sigma-Aldrich) was added to each well. Plates were incubated at 37 °C for 90 min. Bacterial viability was assessed using the previously described colorimetric method with MTT [15,21]. The absorbance (OD) was then measured at a wavelength of 570 nm using a microplate reader (Anthos Labtec Instruments, Wals, Austria). Lysed cell samples (3 μL) from the previous MTT assay were carefully diluted 10-fold in series, 10^−1^ to 10^−6^. These dilutions were then spotted onto appropriate agar plates (Nutrient agar (NA) for Staphylococci and MRS plates for *Weissella paramesenteroides*). Plates were incubated at 37 °C for 18 h to allow for colony growth. Images of the colonies were captured using the GelMax system (Ultra-Violet Products, Cambridge, UK) [21].

### 2.9. Gene Expression Using Real-Time PCR (qPCR)

The effects of minor pathogens in the pathogenesis of bovine mastitis on bovine neutrophils were investigated utilizing gene expression patterns after the cells were exposed to SCH and WPA bacteria. Bovine neutrophils (1 × 10^6^ cells) were placed into 1.5 mL microcentrifuge tubes, and then live bacteria (1 × 10^7^ bacteria) were induced to express genes for 2 h at 37 °C with 5% CO_2_. Control cells were stimulated with HBSS instead of live bacteria. To prepare for RNA extraction, cells were first harvested and washed, then stored in RNA*later* (Invitrogen). Before reverse transcription, the concentration of this RNA was determined using a NanoDrop™ One instrument (Thermo Fisher Scientific, Waltham, MA, USA). Later, 5 µg of this RNA was used for reverse transcription using the Viva cDNA Synthesis Kit (Vivantis Technologies, Selangor Darul Ehsan, Malaysia) following the manufacturer’s instructions. To investigate the expression of genes, 100 ng cDNA samples isolated from bovine neutrophils were analyzed. The analysis focused on three categories: mRNA transcripts of pathogen recognition: *TLR1*, *TLR2*, and *TLR6*; cellular functions—*CYBA* (p22^phox^), *NOX1*, and *SOD1*; and cellular apoptotic genes*—BAX*, *FAS*, *CASP3*, *CASP9, CFLAR* (CASP8 and FADD Like Apoptosis Regulator), *BCL2*, and *BCL2L1* (also called *Bcl-xL*) [15,21]. The real-time RT-PCR (qPCR) reactions were run using a HOT FIREPol^®^ EvaGreen^®^ qPCR Mix Plus (Solis BioDyne, Tartu, Estonia) on a CFX96 Touch Real-Time PCR Detection System (Bio-Rad, Hercules, CA, USA). Gene expression levels normalized to *ACTB* as endogenous controls were calculated using the 2^−ΔΔCt^ method and expressed as mean ± SEM relative to the control condition. The primer information used in the current study is listed in detail in Supplementary Table S1.

### 2.10. Data Analysis

Data normality was assessed using Shapiro–Wilk, Anderson–Darling, and D‘Agostino and Pearson tests. For normally distributed data, one-way ANOVA with Tukey’s or HSD test was used. Otherwise, Kruskal–Wallis with Dunn’s test was performed to compare variations in cellular functions and gene expressions of bovine neutrophils as influenced by minor bovine mastitis-causing bacteria. Data analysis was performed using GraphPad Prism 9.0 or R 4.2.1 with RStudio 2023.12.0. Bubble plots and heatmaps were generated in R using *ggplot2*, *ggfortify*, and *heatmap.2* in *gplots* packages. Significance was set at *p* < 0.05. Data are presented as mean ± SE.

## 3. Results

### 3.1. Bovine Neutrophils Showed Little Response to Minor Pathogens in Terms of Programmed Cell Death Initiation

**Figure 1:**
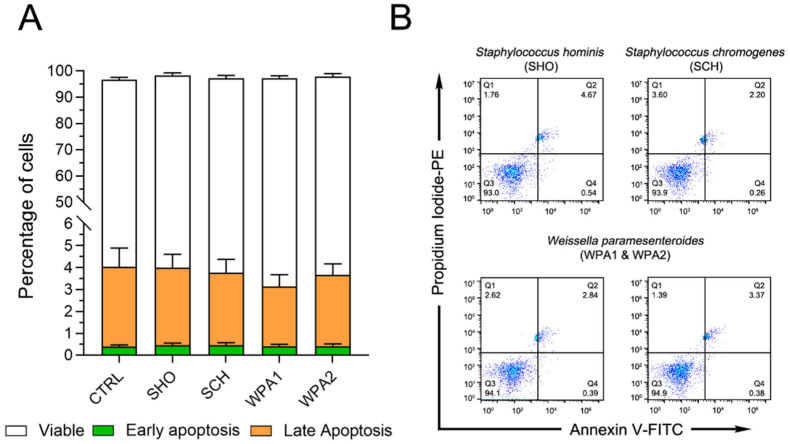
Flow cytometry analysis revealed no effect of NAS or LAB bacteria on bovine neutrophil apoptosis. (**A**) Percentage of viable (Annexin V^−^PI^−^), early-apoptotic (Annexin V^+^PI^−^), and late-apoptotic/dead cells (Annexin V^+^PI^+^) of bovine neutrophils (*n* = 9) stimulated with various NAS and LAB bacteria or left untreated (CTRL). (**B**) Representative flow cytometry data for Annexin V and Propidium Iodide (PI) staining. These data correspond to the results shown in (**A**). Note: NASM = Non-*aureus* staphylococci, LAB = Lactic acid bacteria, CTRL = non-stimulated cells, SHO = *Staphylococcus hominis*, SCH = *Staphylococcus chromogenes*, WPA = *Weissella paramesenteroides*.

This study investigated the effect of minor mastitis-causing bacteria on bovine neutrophil apoptosis using flow cytometry. No significant differences in early (*p* = 0.97, green bars, Annexin V^+^, PI^−^) or late apoptosis (*p* = 0.87, orange bars, Annexin V^+^, PI^+^) were observed between bacteria-exposed and control neutrophils (Figure 1). Additionally, as depicted by the white bars in Figure 1, cell phenotype expression in most groups remained largely in the viable range (Annexin V^−^, PI^−^). Apoptosis remained low across all groups, with early apoptosis values ranging from 0.38% to 0.47%, while late apoptosis values fell between 2.7% and 3.7% (Figure 1). This suggests a minimal impact of bacterial exposure on neutrophil death.

### 3.2. Specific Microbial Associated Molecular Patterns (MAMPs) on Bovine Neutrophils Were Identified and Quantified

**Figure 2:**
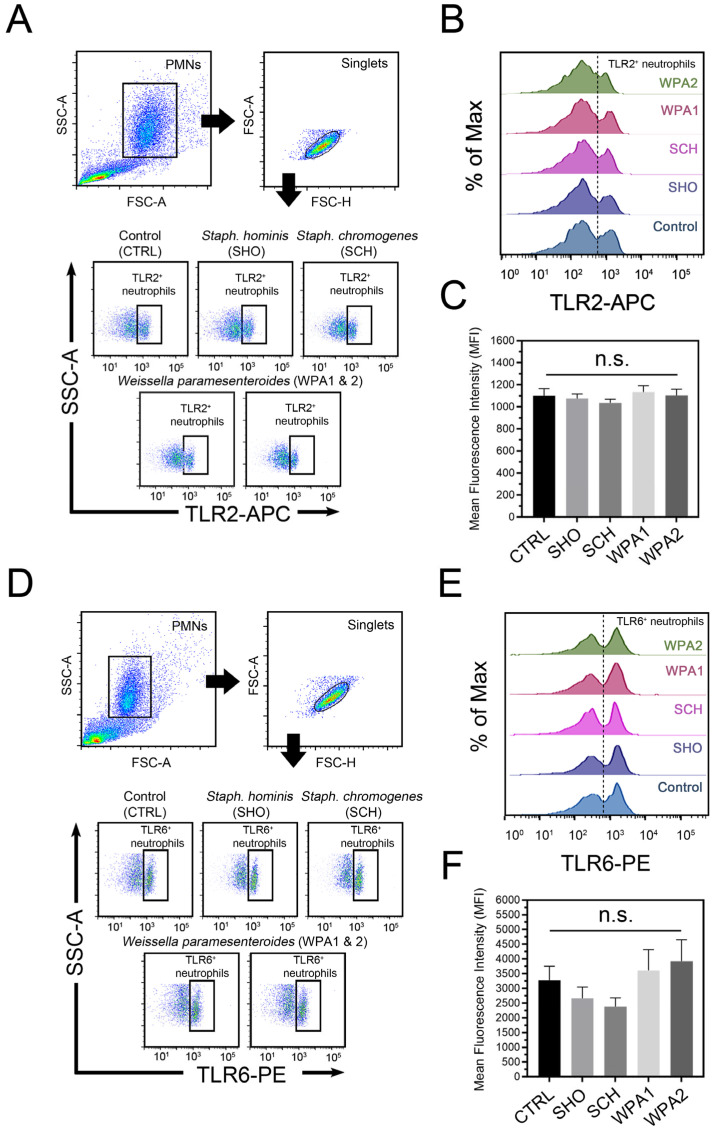
Flow cytometric analysis of microbial recognition receptors on bovine neutrophils stimulated with minor mastitis-causing bacteria. (**A**) Representative flow cytometry dot plot depicting TLR2 surface staining on bovine neutrophils after incubation with SHO, SCH, WPA1, and WPA2 bacteria. (**B**) Half-offset histograms represent the population distribution of bovine neutrophils based on their TLR2 expression levels. Cells exceeding the threshold (dotted line) are considered TLR2-positive. (**C**) Histograms depict mean fluorescence intensity (MFI) for TLR2 expression in TLR2-positive cells across all treatment groups. Data are aggregated from *n* = 7–8 cows. (**D**) Representative flow cytometry analysis of surface TLR6 expression on bovine neutrophils. (**E**) Half-offset histograms depict TLR6-expressing cells (to the right of the dotted line) in each stimulation group. (**F**) Histograms depict MFI for TLR6 expression in TLR6-positive cells across all treatment groups. Data are representative of *n* = 10 cows, n.s. not significant. Note: CTRL = non-stimulated cells, SHO = *Staphylococcus hominis*, SCH = *Staphylococcus chromogenes*, WPA = *Weissella paramesenteroides*.

This study investigated how bovine neutrophils recognize bacterial peptidoglycan and lipoteichoic acid (LTA) using TLR2 and TLR6. While minor bacterial presence modulated TLR2 and TLR6 expression in bovine neutrophils (Figure 2A,B), TLR2 specifically showed minimal response when challenged with various minor mastitis-causing bacteria. When isolated bovine neutrophils encountered a cocktail of individual minor bacteria (SHO, SCH, and both WPA strains), flow cytometry analysis revealed that TLR2, one of the Gram-positive bacteria receptors, remained largely unchanged or constitutively expressed regardless of the bacterial stimulus. Statistical analysis (*p* = 0.51) revealed no significant difference in TLR2 activation (measured by mean fluorescence intensity, MFI) between bovine neutrophils exposed to any minor mastitis-causing bacteria and the unstimulated control group. Mean MFI values across all treatment groups ranged from 1036 to 1134, indicating a minimal impact of bacterial exposure on TLR2 signaling.

Exploring the role of TLR6 in the bovine neutrophil recognition of minor bacteria revealed a complex picture (Figure 2B). Compared to the unstimulated control (MFI = 3278), bovine neutrophils showed reduced TLR6 engagement when challenged with NASM (represented by SHO and SCH), as evidenced by lower mean fluorescence intensity (MFI) values of 2662 and 2384, respectively. Notably, this reduction was not statistically significant (*p* = 0.26). Intriguingly, both WPA strains triggered higher TLR6 expression than NASM or the control. MFI values for WPA1 and WPA2 reached 3606 and 3926, respectively, indicating a stronger utilization of TLR6 for recognition. Our findings paint a fascinating picture of distinctive TLR activation patterns in primed bovine neutrophils upon encountering different minor bacteria (Figure 2). Notably, *Staphylococci* (SHO and SCH) appear to trigger impaired signaling through certain TLRs compared to *Lactobacilli* (WPA1 and WPA2).

### 3.3. Exposure to Minor Mastitis-Causing Pathogens Demonstrated a Modest Magnitude of Intracellular ROS in Bovine Neutrophils

**Figure 3:**
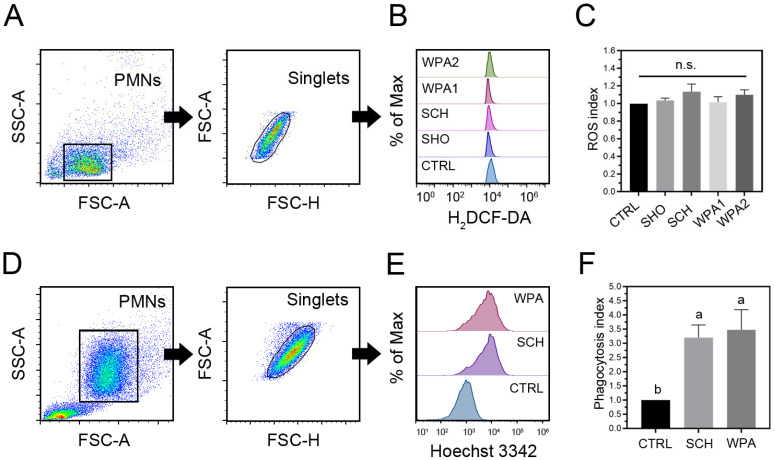
Bovine neutrophils exposed to minor mastitis-causing bacteria show increased reactive oxygen species (ROS) production and phagocytosis. (**A**,**B**) Flow cytometry analysis with gating strategies is employed to quantify intracellular ROS levels in bovine neutrophils. Half-offset histograms are used to visualize the distribution of ROS-positive cells within each treatment group. (**C**) Quantification of intracellular ROS generation in bovine neutrophils (*n* = 10) stimulated with bacteria as ROS index. (**D**,**E**) Flow cytometry analysis demonstrates the phagocytosis of fluorescently labeled minor bacteria by bovine neutrophils. Half-offset histograms represent the distribution of phagocytosis-positive cells across different treatment groups. (**F**) Phagocytic index of bovine neutrophil phagocytosis (*n* = 10) in CTRL, SCH, and WPA groups. Different letters indicate statistically significant differences at *p* < 0.05. Note: n.s. not significant, CTRL = non-stimulated cells, SHO = *Staphylococcus hominis*, SCH = *Staphylococcus chromogenes*, WPA = *Weissella paramesenteroides*.

The effects of minor mastitis bacteria on intracellular ROS production in bovine neutrophils (Figure 3A,C) revealed that all *Staphylococci* strains (SHO, SCH) and LAB strains (WPA1, WPA2) slightly increased ROS production compared to the HBSS control (*p* = 0.12, Figure 3C). This trend, albeit statistically insignificant, suggests a possible low-level activation of the respiratory burst in response to these pathogens. However, looking at the specific fold changes for each group (Figure 3C), we see some subtle variations. SHO exhibited a fold change of 1.04 ± 0.02, representing a very slight increase, while SCH showed a slightly higher fold change of 1.13 ± 0.09, hinting at a potentially stronger interaction with ROS pathways. WPA1 had a fold change of 1.01 ± 0.06, indicating no notable change in ROS production compared to the control and WPA2 displayed a fold change of 1.10 ± 0.06, similar to SCH, suggesting a possible influence on ROS induction. These findings suggest that SCH and WPA2 might elicit a slightly stronger ROS response compared to SHO and WPA1, potentially hinting at differential bacterial recognition by bovine neutrophils. Further studies are needed to validate this hypothesis and explore the underlying mechanisms.

### 3.4. Minor Mastitis Bacteria Substantially Boosted the Ability of Bovine Neutrophils to Engulf the Bacteria

Bovine neutrophils significantly increased the phagocytosis of Hoechst 33,342 labeled SCH and WPA2 bacteria compared to controls (*p* = 0.002, Figure 3F). These data demonstrate a heightened internalization capacity of neutrophils towards minor mastitis-causing bacteria, as evidenced by fold changes of 3.20 ± 0.44 for SCH and 3.48 ± 0.71 for WPA2, respectively (Figure 3E,F).

### 3.5. Staphylococcus Chromogenes Exhibited Potential Resistance to the Bactericidal Activity of Bovine Neutrophils

**Figure 4:**
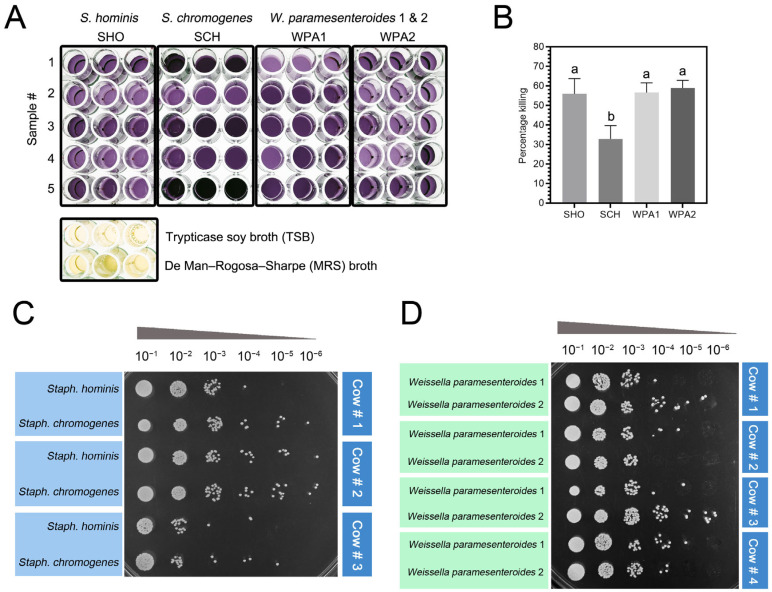
*Staphylococcus chromogenes* exhibited resistance to bovine neutrophil killing as measured by the MTT assay. (**A**) Representative image of formazan formed by bacteria in a 96-well plate, following an MTT assay. The color intensities were used to quantify the percentage killing of SHO, SCH, WPA1, and WPA2 compared to blank wells containing Tryptic Soy Broth (TSB) and De Man, Rogosa, and Sharpe (MRS) media. (**B**) The histograms illustrate the mean percentage killing of minor bacteria in each treatment (*n* = 9). Different letters indicate statistically significant differences at *p* < 0.05. (**C**,**D**) Representative image of bacterial colonies recovered from bovine neutrophil lysate following 10-fold serial dilutions. The colonies were derived from an MTT assay and spotted onto nutrient agar (NA) plates (**C**) or MRS agar plates (**D**) for 18 h at 37 °C incubation. Note: SHO = *Staphylococcus hominis*, SCH = *Staphylococcus chromogenes*, WPA = *Weissella paramesenteroides*.

We investigated the ability of bovine neutrophils to combat minor mastitis pathogens by co-incubating them with either *Staphylococci* (NASM) or lactic acid bacteria (LAB) and measuring bacterial viability and proliferation through the MTT and spot dilution assay (Figure 4). Bovine neutrophils reduced the viability of *Staphylococcus hominis* (SHO) by 55.98% ± 7.79% and *Staphylococcus chromogenes* (SCH) by 32.83% ± 6.79%. Interestingly, both lactic acid bacteria (LAB) strains—*Weissella paramesenteroides* WPA1 and WPA2—displayed higher susceptibility to neutrophil activity, with reductions of 56.61% ± 4.92% and 58.96% ± 3.85%, respectively (Figure 4B). One-way ANOVA analysis of the MTT assay data revealed a significant difference (*p* = 0.01, Figure 4B) in the overall effectiveness of bovine neutrophils against minor mastitis-causing bacteria. This difference primarily stemmed from SCH exhibiting a lower level of susceptibility to neutrophil killing compared to the other bacteria studied, including both NASM and LAB strains.

### 3.6. Minor Mastitis Bacteria Significantly Upregulated Gene Expression in Pathways Related to Bacterial Recognition, ROS Production, and Cellular Apoptosis Pathways

**Figure 5:**
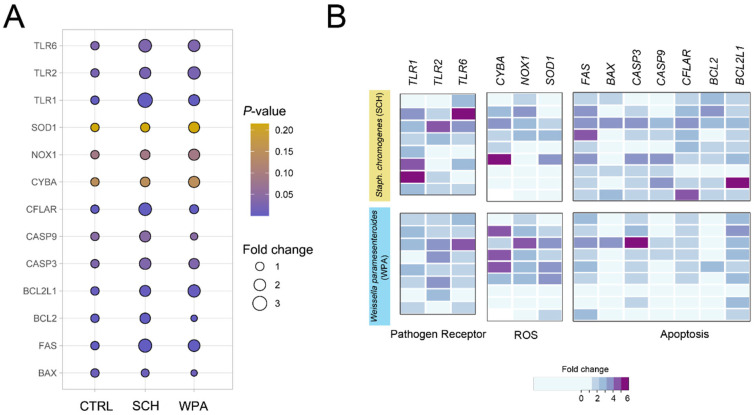
Exposure to both *Staphococcus chromogenes* and *Weissella paramesenteroides* leads to an upregulation of genes associated with pathogen recognition, reactive oxygen species (ROS) generation, and apoptosis in bovine neutrophils. (**A**) A bubble plot depicts the changes in gene expression in bovine neutrophils after stimulation with two minor bacteria (SCH and WPA) compared to non-stimulated controls (CTRL). Each bubble represents a gene involved in either pathogen recognition (*TLR1*, *2*, *6*), ROS generation (*CYBA*, *NOX1*, *SOD1*), or apoptosis (*BAX*, *FAS*, *CASP3*, *CASP9*, *CFLAR*, *BCL2*, *BCL2L1*). (**B**) Heatmaps depict the individual expression profiles of genes identified in (**A**) across bovine neutrophil samples. Each sample was stimulated with SCH or WPA or was not stimulated (CTRL). Expression of CTRL was normalized to a value of 1 and excluded from the visualization. Each column represents an individual sample array (*n* = 8–9), and each row represents a specific gene. The color scale encodes the fold change. Note: CTRL = non-stimulated cells, SCH = *Staphylococcus chromogenes*, WPA = *Weissella paramesenteroides*.

We have studied gene expression in bovine neutrophils after exposure to *Staphylococcus chromogenes* (SCH) and *Weissella paramesenteroides* (WPA2) (Figure 5). We focused on genes encoding key pathogen recognition receptors (TLRs) involved in Gram-positive bacteria detection (*TLR1*, *TLR2*, *TLR6*). Interestingly, both bacteria triggered the upregulation of all three TLRs. Stimulation with SCH elicited a substantial upregulation of the TLR1, TLR2, and TLR6 genes in bovine neutrophils, indicating a strong activation of these key pathogen recognition receptors. Notably, while TLR2 and TLR6 also exhibited significant upregulation upon exposure to lactic acid bacteria (LAB), TLR1 expression remained markedly lower compared to SCH, suggesting a potentially differential mechanism of recognition for this specific pathogen. The significant upregulation of these TLR genes in neutrophils stimulated with NASM and LAB confirms their key function in this process. Detailed expression levels compared to unstimulated controls are presented in Table 1 and Figure 5.

Beyond pathogen recognition genes, we found genes crucial for bacterial destruction, including those encoding ROS-generating enzymes (*CYBA*, *NOX1*, *SOD1*) used by neutrophils for phagocytosis and intracellular killing. We investigated key genes in the NOX family (*CYBA* or p22^phox^ subunit, *NOX1*, *SOD1*) for their role in pathogen damage. These genes encode enzymes forming ROS, with *SOD1* converting harmful superoxide anions (O_2_^−^) to less-harmful hydrogen peroxide (H_2_O_2_) and molecular oxygen (O_2_). Real-time PCR analysis showed slight upregulation in all genes (Table 1) but only *NOX1* had a significant difference (*p* = 0.08) for SCH and WPA2, aligning with flow cytometry data suggesting a more prominent role for NOX1 in ROS production. Examining apoptosis genes in response to bacteria, we found pro-apoptotic *FAS* to be upregulated (*p* = 0.0005, Table 1) in neutrophils, while *BAX* was downregulated (*p* = 0.0097), suggesting a complex regulation of cell death.

Within the realm of apoptotic signaling, caspases play crucial roles, categorized as either initiator caspases (caspases 8 and 9), responsible for initial activation, or executioner caspases (caspases 3, 6, and 7), driving downstream effector functions. Investigating caspases in response to bacteria, we found initiator caspase *CFLAR* to be upregulated (*p* = 0.0011) in both *Staphylococcus chromogenes* (SCH) and lactic acid bacteria (LAB)-stimulated neutrophils (*p* = 0.0011, Table 1), suggesting its role in the response. While the *CASP9* response varied between bacteria (*p* = 0.044, Table 1), *CASP3* (executioner caspase) was upregulated in both groups, suggesting complex caspase regulation (*p* = 0.032, Table 1). This finding supports the potential for *CASP3* to hold a central position in the apoptotic process triggered by these minor mastitis-causing bacteria compared to the observed responses of initiator caspases like *CASP8* and *CASP9*. Although *CASP3*, *CFLAR*, and *CASP9* are all classified as executioner caspases and potentially contribute to the apoptotic response of bovine neutrophils to NASM bacteria, this study suggests that only *CASP3* and *CFLAR* participate in the initiation of apoptosis when these cells encounter LAB bacteria.

Pro-survival members of the Bcl-2 family, like Bcl-2 and Bcl-xL, can suppress caspase activation and pro-apoptotic processes, influencing the overall apoptotic response. *BCL2* and *BCL2L1* were upregulated in SCH-stimulated neutrophils (*p* < 0.0001 and *p* = 0.0035, respectively, Table 1), suggesting the modulation of apoptosis. Unlike cells stimulated with SCH, cells in the LAB group downregulated *BCL2* but upregulated *BCL2L1*, similar to the SCH response. Comparing the expression patterns of pro-apoptotic and anti-apoptotic genes in bovine neutrophils challenged with SCH and LAB bacteria reveals their contrasting roles in triggering cell death. SCH likely acts as a potent apoptotic stimulus, upregulating both pro-apoptotic pathways (*FAS*, *CFLAR*, *CASP9*, and *CASP3*) and anti-apoptotic genes (*BCL2* and *BCL2L1*). In contrast, the LAB strain appears to be a weaker apoptotic inducer, activating only one upstream initiator caspase (*CFLAR*) and one anti-apoptotic gene (*BCL2L1*).

## 4. Discussion

Non-*aureus* staphylococci and mammaliicocci (NASM) are gaining attention for causing subclinical mastitis because certain NASM strains can cause damage to the udder tissues, leading to intramammary infections [5]. The presence of multiple genes resistant to antibiotics commonly used in dairy settings, including penicillin (*bla*Z), methicillin (*mecA* and *mec*C), tetracycline (*tet*K and *tet*M), and erythromycin (*erm*A, *erm*B, and *erm*C), alongside other virulence factors in this type of bacteria, has sparked debate among researchers [9]. Bovine neutrophils displayed minimal apoptotic phenotype expression upon exposure to minor mastitis bacteria, aligning with flow cytometry data and suggesting little effect on apoptosis induction [15]. However, a recent report suggested that the dosage used for in vitro bacterial infection can significantly suppress neutrophil functions and cell death [22]. Though using an in vitro MOI of 10 aligns with prior studies, future experiments should explore diverse dosages for a more definitive understanding of how minor mastitis bacterial exposure impacts apoptosis. Minor mastitis-causing bacteria may not trigger significant neutrophil apoptosis, potentially prioritizing other defenses like phagocytosis or tailoring responses to pathogenic bacterial threats [23]. Previous work suggests stronger apoptosis with major mastitis pathogens (*Staphylococcus aureus*, and *Streptococcus uberis*) compared to the minor ones used here (*Staphylococcus hominis*, *Staphylococcus chromogenes*, and LAB). This study’s bacteria may prioritize neutrophil defense mechanisms rather than apoptosis [15].

The timing of analysis plays a crucial role, as early apoptotic events might be transient and challenging to detect at specific moments. Investigating apoptosis at different time points could provide additional insights [24]. Although exposure to TLR ligands did not trigger apoptosis in bovine PMNs [25], their involvement in inflammasome activation and subsequent silent cell death through pyroptosis cannot be ruled out [19]. Investigating other cell death pathways, like necrosis, NETosis, and pyroptosis, could be informative as bovine neutrophils might employ alternative mechanisms against minor pathogens [15,20,26].

Specific microbial-associated molecular patterns (MAMPs) on bovine neutrophils were previously extensively identified and quantified [27,28]. Here, we reveal distinct patterns of Toll-like receptor (TLR) activation in response to minor mastitis-causing bacteria. Exploring the role of TLR6 in the bovine neutrophil recognition of NASM and LAB revealed a complex pattern. Bovine neutrophils showed reduced TLR6 engagement when challenged with NAS, represented by *S. hominis* and *S. chromogenes*. On the contrary, LAB strains (*Weissella paramesenteroides*) induced significantly higher TLR6 expression. These findings suggest that different pathogens may utilize distinct MAMPs and TLRs for recognition by bovine circulating/milk neutrophils, potentially influencing the subsequent immune response [16,25,27]. While bovine neutrophils might have evolved specific recognition mechanisms for different levels of pathogenic threat, mutations in the coding regions of *TLR2*, *4*, and *6* genes elevated mastitis susceptibility, suggesting a crucial genetic link to resistance [29,30]. The minimal TLR2 response to minor pathogens could indicate alternative pathways for the recognition and/or prioritization of other defense mechanisms like phagocytosis, as well as the mitigation of apoptosis [25,31].

It has been suggested that the activation of MAPK and the Caspase-1 signaling pathways may involve TLR2, TLR4, and NLRP3 receptors [19]. The observed differences in TLR6 activation between NAS and LAB bacteria could be attributed to their unique MAMP profiles. NAS might possess ligands that do not require TLR6 signaling, while LAB might express ligands that enhance it. Supporting the idea that TLR2 relies on TLR6 as a co-receptor for diacylated lipopeptide recognition [32], research on lipoprotein-deficient bacteria reveals a lower expression of the TLR2 gene compared to wild-type bacteria [19]. The SNPs were identified among TLR2, 4, and 6 in mastitis-susceptible animals, with mutations in TLRs that were repeatedly reported in susceptible cows [29]. This study suggests that TLRs play a role in how bovine neutrophils respond to minor mastitis bacteria. Future work on TLR-triggered downstream signaling pathways (inflammation, apoptosis) could reveal how these receptors influence the immune response to minor mastitis pathogens [31].

Minor mastitis bacteria triggered modest ROS production in bovine PMNLs, aligning with previous findings for *S. chromogenes* [33]. Bovine neutrophils appear to tailor their response based on the severity of the challenge. Minor mastitis-causing bacteria like *S. chromogenes* and *W. paramesenteroides* may trigger a low-level, non-specific ROS burst instead of a full-blown oxidative response reserved for highly virulent pathogens, such as *S. aureus* [33]. Variations in bacteria-induced ROS suggest complex interplays and potential differences in how neutrophils recognize these minor mastitis pathogens. Further investigation into underlying mechanisms and potential differential recognition pathways is needed.

In line with previous reports, bovine neutrophils may favor phagocytosis as the primary defense against minor mastitis-causing bacteria such as *W. paramesenteroides* [15]. This efficient internalization could effectively neutralize and clear the pathogens before they cause significant inflammation or damage. Though minor pathogens often fail to induce strong apoptosis or ROS production (as previously discussed), bovine neutrophils readily utilize phagocytosis against both *S. aureus* and NAS bacteria [33,34]. However, *Staph. aureus* demonstrates greater resistance to killing within neutrophils, compared to *S. chromogenes* [34]. The observed differences in internalization between NAS and LAB suggest potential variations in their pathogen recognition by neutrophils or susceptibility to phagocytic mechanisms. Further investigation into specific MAMPs and the host receptors involved could elucidate these differences.

Understanding the fate of internalized bacteria necessitates exploring their potential for degradation within phagolysosomes or the existence of escape mechanisms. *S. chromogenes* displayed potential resistance to the bactericidal activity compared to other minors in this study. A study by Kampen et al. found that the capsular polysaccharides (CPS) of *S. aureus* protect the bacteria from being engulfed and killed by bovine neutrophils while also reducing the production of ROS [35]. Evidence suggests that bacteria evade neutrophil engulfment with various strategies like surface proteins, CPS, or biofilms that mask opsonization sites or interfere with neutrophil engulfment [36,37,38]. This aligns with our findings, suggesting that *S. chromogenes* might possess unique mechanisms to resist intracellular killing by bovine neutrophils. Comparing *S. chromogenes* to other *Staphylococci* strains could identify factors influencing resistance. Additionally, investigating the long-term effects of its persistence in neutrophils might link it to chronic mastitis development [6].

Cells exposed to *S. chromogenes* and *W. paramesenteroides* showed increased activity in genes involved in recognizing Gram-positive bacteria (*TLR1*, *2*, *6*) and producing ROS (*CYBA*, *NOX1*, *SOD1*). The genes associated with cell death pathways exhibited varied expression. Cells exhibit a robust initial response to minor mastitis-causing bacteria through the upregulation of all TLRs tested, potentially as a first line of pathogen detection. Differences in TLR expression patterns observed between those bacteria suggest distinct MAMP profiles or recognition mechanisms employed [39,40]. These observations highlight the versatility of neutrophil responses, emphasizing their ability to adapt to varying threats based on the MAMP profile of the bacteria they encounter. Despite observing upregulated apoptosis genes, their functional importance in neutrophil–pathogen interactions with minor pathogens remains unclear. Further investigation is necessary to understand if these changes translate to altered apoptotic phenotypes or other functional consequences.

The current study showed that bovine neutrophil exposure to minor mastitis-causing bacteria resulted in the upregulation of genes associated with key components involved in ROS generation, with a specific focus on *NOX1*, *CYBA* (p22^phox^), and *SOD1* potentially playing a prominent role in this response [33]. Upregulation of *NOX1* suggests the priming of the NADPH oxidase complex for potential ROS production in response to minor mastitis-causing bacteria. This might represent a strategic response where neutrophils prioritize other defense mechanisms (e.g., phagocytosis) initially and hold the full respiratory burst in reserve for potential escalation if needed [33]. The observed variation in gene expression across *CYBA*, *NOX1*, and *SOD1* could reflect a fine-tuned ROS generation process where different enzymes are used depending on the specific bacterial challenge and desired ROS type (e.g., superoxide vs. hydrogen peroxide).

Given the persistence of subclinical intramammary infections (pIMI) caused by non-*aureus* staphylococci (NAS) [6,41], it is possible that bovine neutrophils utilize diverse apoptotic strategies as part of their immune response to combat these bacterial strains. According to our findings, *S. chromogenes* showed a prominent upregulation of genes involved in both apoptosis and anti-apoptosis in bovine neutrophils and exhibited a contrasting apoptotic phenotype. Cells strategically modulate their pro-apoptotic response towards minor pathogens. This allows them to limit excessive self-damage, prioritizing alternative defense pathways and conserving resources for more significant threats. The simultaneous upregulation of both pro-apoptotic and anti-apoptotic genes/proteins in bovine neutrophils hints at intricate regulatory mechanisms within their apoptotic network, suggesting tightly controlled and multifaceted responses [18,21]. The downregulation of *BAX* by cells exposed to either *S. chromogenes* or *W. paramesenteroides*, despite the upregulation of other pro-apoptotic genes, indicates additional regulatory roles beyond the Bcl-2 family in bovine neutrophil apoptosis. The ability to manipulate apoptosis within bacteria-harboring neutrophils could be essential for maintaining the cellular longevity, viability and functionality of neutrophils, enabling them to effectively combat these sustained challenges [22,42].

Other pro- and anti-apoptotic proteins or signaling pathways might be involved in fine-tuning the response. Exploring the downstream consequences of apoptosis induction, such as the release of pro-inflammatory mediators or phagocytosis of apoptotic cells, would offer insights into its role in the overall immune response to minor mastitis-causing pathogens.

## 5. Conclusions

This study sheds new light on the role of minor pathogens like NASM and LAB in bovine mastitis, challenging the traditional perception of their insignificance. While LAB strains triggered minimal neutrophil activation and apoptosis, *Staphylococcus chromogenes* displayed a distinct strategy. It effectively evaded neutrophil-killing mechanisms by downregulating TLR6 recognition and weakening the phagocytic response. Interestingly, *S. chromogenes* also induced a unique gene expression pattern, suggesting its ability to manipulate neutrophil functions. These findings highlight the multifaceted nature of NASM involvement in mastitis, prompting a re-evaluation of their pathogenic potential and the development of targeted strategies to combat them. Further research into the specific mechanisms employed by *S. chromogenes* could pave the way for novel therapeutic approaches to tackle this emerging mastitis threat.

## Figures and Tables

**Table 1:** Results of the one-way ANOVA/Kruskal–Wallis test, and Tukey’s Multiple Comparison Tests of mRNA expression of bovine neutrophil-associated genes by real-time PCR performed on bovine neutrophils stimulated with minor mastitis-causing pathogens.

Gene	CTRL	SCH	WPA	*p* Value	Test
*TLR1*	1.00 ± 0.00 ^b^	3.22 ± 0.54 ^a^	1.79 ± 0.29 ^b^	0.001	ANOVA
*TLR2*	1.00 ± 0.00 ^b^	1.89 ± 0.44 ^ab^	2.31 ± 0.32 ^a^	0.023	ANOVA
*TLR6*	1.00 ± 0.00 ^b^	2.45 ± 0.50 ^a^	2.09 ± 0.39 ^ab^	0.030	ANOVA
*CYBA*	1.00 ± 0.00 ^a^	1.89 ± 0.54 ^a^	2.19 ± 0.51 ^a^	0.148	ANOVA
*NOX1*	1.00 ± 0.00 ^a^	1.36 ± 0.32 ^a^	1.84 ± 0.30 ^a^	0.088	ANOVA
*SOD1*	1.00 ± 0.00 ^a^	1.21 ± 0.32 ^a^	1.78 ± 0.36 ^a^	0.214	Kruskal–Wallis
*FAS*	1.00 ± 0.00 ^b^	2.63 ± 0.47 ^a^	2.07 ± 0.29 ^ab^	0.0005	Kruskal–Wallis
*BAX*	1.00 ± 0.00 ^a^	0.89 ± 0.27 ^a^	0.54 ± 0.14 ^b^	0.0097	Kruskal–Wallis
*CFLAR*	1.00 ± 0.00 ^b^	2.44 ± 0.41 ^a^	1.17 ± 0.18 ^b^	0.0011	ANOVA
*CASP3*	1.00 ± 0.00 ^b^	1.98 ± 0.36 ^a^	1.58 ± 0.24 ^ab^	0.0318	ANOVA
*CASP9*	1.00 ± 0.00 ^ab^	1.81 ± 0.44 ^a^	0.74 ± 0.23 ^b^	0.0444	Kruskal–Wallis
*BCL2*	1.00 ± 0.00 ^b^	1.61 ± 0.21 ^a^	0.56 ± 0.11 ^b^	<0.0001	ANOVA
*BCL2L1*	1.00 ± 0.00 ^b^	1.78 ± 0.30 ^ab^	2.29 ± 0.31 ^ab^	0.0035	ANOVA

Note: CTRL = non-stimulated cells, SCH = *Staphylococcus chromogenes*, WPA = *Weissella paramesenteroides*. The values are mean ± SEM, any means within a row with shared superscript letters are not significantly different from each other at *p* < 0.05 with Tukey’s multiple comparison test.

## Data Availability

Data are contained within the article and Supplementary Materials.

## Supplementary Materials

The following supporting information can be downloaded at: https://www.mdpi.com/article/10.3390/vetsci11060262/s1, Table S1: List of primer sequences and related information used in this study.
