# Supplementary material for: Divergent Immune Responses to Minor Bovine Mastitis-Causing Pathogens
Source: Vet Sci. 2024 Jun 7;11(6):262. doi: 10.3390/vetsci11060262 (PMC11209595; doi:10.3390/vetsci11060262)
Supplement: Supplementary file 1 [file vetsci-11-00262-s001.zip › vetsci-2990942-supplementary.pdf]

Supplementary Table S1.

List of primer sequences and related information used in this study

Gene	Accession No.	Primer	Sequence	Size (bp)	Reference
CYBA	NM_174034	Forward	TCAGTTCACCCAGTGGTACC	135	[21]
		Reverse	ACTCTGGTCAGGTACTTCTGTC		
NOX1	NM_001191340	Forward	TGTCTGGGGTCAAACAGAAGAG	112	[15]
		Reverse	TTCAAATTGGGGAGGCTTGC		
SOD1	NM_174615.2	Forward	GCTGACAAAAACGGTGTTGC	131	[15]
		Reverse	TCATTTCCACCTCTGCCCAAG		
TLR1	NM_001046504	Forward	ACTTTGTTGCTGGCAAGAGC	101	[15]
		Reverse	TTCGCTCTGGACAAAGTTGG		
TLR2	XM_005217447.4	Forward	TGCTGCCATTCTGATTCTGC	138	[15]
		Reverse	AACCAAAACCCTTCCTGCTG		
TLR6	NM_001001159	Forward	AACTTTGTTGCCGGCAAGAG	103	[15]
		Reverse	ACTCGCTCTGGACAAAGTTG		
BAX	NM_173894	Forward	TCGCCCTTTTCTACTTTGCC	104	[15]
		Reverse	TCGAAGGAAGTCCAATGTCCAG		
FAS	NM_174662.2	Forward	GAAAATGCCCACATGGCTGG	140	[15]
		Reverse	TCACCGTTTTTCCGTTTGCC		
BCL2	NM_001166486	Forward	GTATGGCCCTAGCATGCGG	116	[15]
		Reverse	ACTTATGGCCCAGATAGGCA		
BCL2L1	NM_001077486	Forward	TGTGGCCTTTTTCTCCTTCG	108	[15]
		Reverse	TCATTCAGGTAAGTGGCCATCC		
CASP3	NM_001077840	Forward	AGTGGTGCTGAGGATGACATG	119	[15]
		Reverse	AACCAGGATCCGTTCTTTGC		
CASP9	NM_001205504.2	Forward	GAGTCAGGCCCTTCCTTTGTT	115	[15]
		Reverse	CGGCTTTGATGGGTCATCCT		
CFLAR	NM_001012281	Forward	ACTCACTCTGGGGTCCCTTT	122	[15]
		Reverse	AGCTGGCCCTCTGATTTCAC		
ACTB	NM_173979	Forward	TGCGGCATTCACGAAACTAC	146	[15]
		Reverse	AGGGCAGTGATCTCTTTCTGC		
